# TERRA G-quadruplex stabilization as a new therapeutic strategy for multiple myeloma

**DOI:** 10.1186/s13046-023-02633-0

**Published:** 2023-03-27

**Authors:** Francesca Scionti, Giada Juli, Roberta Rocca, Nicoletta Polerà, Matteo Nadai, Katia Grillone, Daniele Caracciolo, Caterina Riillo, Emanuela Altomare, Serena Ascrizzi, Basilio Caparello, Maria Cerra, Mariamena Arbitrio, Sara N. Richter, Anna Artese, Stefano Alcaro, Pierosandro Tagliaferri, Pierfrancesco Tassone, Maria Teresa Di Martino

**Affiliations:** 1 Department of Experimental and Clinical Medicine, Magna Graecia University, Catanzaro, Italy; 2 Net4science Srl, Università degli Studi “Magna Graecia” di Catanzaro, Catanzaro, Italy; 3 Department of Molecular Medicine, University of Padua, Via A. Gabelli 63, 35121 Padua, Italy; 4 Presidio Ospedaliero “Giovanni Paolo II”, Lamezia Terme, Catanzaro, Italy; 5 Institute of Research and Biomedical Innovation (IRIB), Italian National Council (CNR), 88100 Catanzaro, Italy; 6 Department of Health Sciences, Magna Graecia University of Catanzaro, Campus “Salvatore Venuta”, Viale Europa, 88100 Catanzaro, Italy

**Keywords:** Multiple myeloma, TERRA, Telomere dysfunction, TRF2, Small molecules, DNA damage, Genomic instability, Long non-coding RNA, lncRNAs

## Abstract

**Background:**

Multiple myeloma (MM) is a hematologic malignancy characterized by high genomic instability, and telomere dysfunction is an important cause of acquired genomic alterations. Telomeric repeat-containing RNA (TERRA) transcripts are long non-coding RNAs involved in telomere stability through the interaction with shelterin complex. Dysregulation of TERRAs has been reported across several cancer types. We recently identified a small molecule, *hit* 17, which stabilizes the secondary structure of TERRA. In this study, we investigated in vitro and in vivo anti-MM activities of *hit* 17.

**Methods:**

Anti-proliferative activity of *hit* 17 was evaluated in different MM cell lines by cell proliferation assay, and the apoptotic process was analyzed by flow cytometry. Gene and protein expressions were detected by RT-qPCR and western blotting, respectively. Microarray analysis was used to analyze the transcriptome profile. The effect of *hit* 17 on telomeric structure was evaluated by chromatin immunoprecipitation. Further evaluation in vivo was proceeded upon NCI-H929 and AMO-1 xenograft models.

**Results:**

TERRA G4 stabilization induced in vitro dissociation of telomeric repeat‐binding factor 2 (TRF2) from telomeres leading to the activation of ATM-dependent DNA damage response, cell cycle arrest, proliferation block, and apoptotic death in MM cell lines. In addition, up-regulation of TERRA transcription was observed upon DNA damage and TRF2 loss. Transcriptome analysis followed by gene set enrichment analysis (GSEA) confirmed the involvement of the above-mentioned processes and other pathways such as E2F, MYC, oxidative phosphorylation, and DNA repair genes as early events following *hit* 17-induced TERRA stabilization. Moreover, *hit* 17 exerted anti-tumor activity against MM xenograft models.

**Conclusion:**

Our findings provide evidence that targeting TERRA by *hit* 17 could represent a promising strategy for a novel therapeutic approach to MM.

**Supplementary Information:**

The online version contains supplementary material available at 10.1186/s13046-023-02633-0.

## Background

Multiple myeloma (MM) is a plasma cell malignancy characterized by high genomic instability [1] that leads to progressive accumulation of genomic alterations which occur starting from pre-malignant stages of monoclonal gammopathy of undetermined significance (MGUS) and smoldering MM (SMM) to clinical overt MM [2]. Telomere dysfunction is an important source of genomic instability [3]. Telomeres are loop structures localized at chromosome ends and consisting of six nucleotide repetitive sequences (5′-TTAGGG-3′) associated with a group of shelterin proteins [4]. The maintenance of telomere architecture is crucial to prevent its targeting by DNA damage repair machinery and chromosome end-to-end fusion or structural rearrangements at chromosome ends. Telomeric repeat-containing RNA (TERRA) transcripts are a class of long non-coding RNAs (lncRNAs) transcribed by RNA polymerase II (RNAPII) from the sub-telomeric to the partial telomeric regions of different chromosomes [5]. These lncRNAs are known to form G-quadruplex (G4) secondary structures which contain square planar alignment of four guanines connected by Hoogsteen hydrogen bonds and stabilized by a monovalent cation such as K^+^ and Na^+^ [6]. TERRA G4 actively participates in genome stability by interacting with the glycine-arginine rich (GAR) domain of the shelterin protein TRF2 [7, 8] that is required for t-loop formation and maintenance [9]. TERRA transcripts are also negative regulators of human telomerase [10] and contribute to heterochromatin formation by their interaction with the histone methyltransferase Polycomb repressive complex 2 (PRC2) [11]. Transcriptional dysregulation of TERRA has been reported across several cancer types although with different trends. Notably, deregulation of TERRA has been observed in some studies, thus indicating that TERRA transcripts may act as tumor suppressors [12–14]. However, the role of TERRA in MM is still unclear. In the last years, one approach used for the investigation of lncRNAs in human cancer was based on the design of small molecules that are directed toward their secondary structures [15]. In particular, the targeting of telomeric components with G4 ligands has emerged as a therapeutic approach in human malignancies [16–19]. We recently reported that a benzofuran derivative named *hit* 17, identified by a virtual screening of small molecule libraries, can physically interact and stabilize TERRA G4 [20, 21]. To explore the functional role of TERRA in MM, we investigated in vitro and in vivo activity of the small molecule *hit* 17.

## Methods

### Chemical compound

*Hit* 17 was obtained by a virtual screening on TERRA and DNA G4 and purchased from InterBioScreen (InterBioScreen Ltd). Dry powered was dissolved in dimethyl sulfoxide (DMSO) at concentration of 10 mM and stored at -20 °C in small aliquots. For in vivo experiments, *hit* 17 saline solutions were used in one week, stored at -20 °C and avoid more than two defrost cycles.

### Cell cultures

NCI-H929, RPMI-8226, AMO-1, and AMO-BZB MM cell lines were purchased from DSMZ, certified authentication performed by short tandem repeat DNA typing, and were cultured in RPMI-1640 media containing 10% fetal bovine serum (FBS) (Corning, Tewksbury MA, USA), 2 μmol/L glutamine, 100 U/mL penicillin, and 100 μg/mL streptomycin (Sigma Aldrich, St Louis, MO, USA) at 37 °C in a 5% CO_2_ atmosphere. Peripheral blood mononuclear cells (PBMCs) were isolated by Ficoll-hypaque (Lonza Group, Basel, Switzerland) following the manufacturer’s protocol.

### Analysis of cell viability and apoptosis

Cell viability was evaluated by Cell Titer-Glo assay (Promega, Madison, WI, USA) according to manufacturer’s protocol. Apoptosis was assessed by Attune^TM^NxT Flow cytometer (Thermo Fisher Scientific, Inc.) following annexin V-7-aminoactinomycin D (7AAD) staining (BD Pharmingen).

### Western blotting (WB) analysis and antibodies

Whole cell protein extracts were prepared using NP40 lysis buffer containing Halt Protease Inhibitor cocktail (Thermo Fisher Scientific, Inc.), separated using 4–12% or 3–8% Nupage Bis–Tris SDS-acrylamide gels (Thermo Fisher Scientific, Inc.), and electro-transferred on nitrocellulose membranes (Bio-Rad) by a Trans-Blot® Turbo™ Transfer Starter System for 30 min. Nitrocellulose membranes were blocked with milk and probed over-night with primary antibodies at 4 °C, then membranes were washed 3 times in phosphate buffered saline with Tween^TM^ 20 (PBS-Tween), incubated with a secondary antibody conjugated with horseradish peroxidase for 2 h at room temperature. Chemiluminescence was detected using SuperSignal West Pico PLUS Substrate (Thermo Fisher Scientific, Inc.) and visualized with a C-DiGit® Blot Scanner (LI-COR, version 5.0). WB was performed using Cell Signaling antibodies: p-ATM (#5883S), ATM (#2873S), p-CHK1 (#2348S), p-CHK2 (#2197S), CASPASE-3 (#9665S), p21 (#2946S), H3 (#4499S), H3K9me3 (#5327S), H3K27me3 (#9733S), H4K20me3 (#5737S), H4 (#13919S), p-γH2AX (#9718S), RAD51 (#8875S). GAPDH (sc-25778), Actin (sc-58673), PARP1 (sc-8007), TERT (sc-377511), and LIGASE-I (sc-390235) were from Santa Cruz Biotechnology (Dallas, TX, USA), TRF2 (ab13579) was from Abcam. Secondary antibody with fluorochrome conjugate used for immunofluorescence (IF) were: Alexa Fluor 488 goat anti-mouse IgG (H + L), Alexa Fluor 488 goat anti-rabbit IgG (H + L).

### IF and Telomere dysfunction-Induced Foci (TIF) with Cy3-labeled probe

Cells were harvested, centrifuged onto glass slides (Cytospin 4, Thermo Scientific), and fixed in 4% paraformaldehyde in PBS, pH 7.4, for 12 min at 22 °C, followed by three 5-min washes in PBS. After that, cells were permeabilized (0.1% Triton X-100 in PBS, 15-min), washed in PBS (3x, 5 min each), and incubated 1 h at room temperature with blocking buffer (1.5% BSA in PBS). Cells were reacted > 12 h at 4 °C with primary antibodies, washed in PBS (3x, 5 min each), and incubated 1 h at room temperature in the dark, with appropriate secondary antibodies. For IF with Cy3-labeled probe, cells were washed with PBS for 15 min and again fixed with 2% paraformaldehyde at room temperature for 10 min after the hybridization of primary and secondary antibodies. After that cells were dehydrated with ethanol series (70, 95, and 100% for 1 min) and air dried. Cells were then incubated at 80 °C for 5 min with a Cy3-labeled telomeric FISH probe (Panagene). Then the coverslips were transferred to 4 °C for overnight. Next day, coverslips were washed with wash buffer (10 mM Tris HCl, pH 7.4) containing 50% (v/v) formamide at 50 °C for 15 min. Then a second wash was done for 5 min using PBS. Cells were then mounted with Vectashield with DAPI (Vector Laboratories). Images were collected on a Leica DM4 B Upright Microscope (Leica Microsystems), using a HC FL PLAN 40x/0.65 objective. TIF was detected by IF using γ-H2AX foci in combination with telomere PNA probe.

### Reverse transcription quantitative PCR (RT-qPCR)

Total RNA was extracted from cells using TRIzol® reagent (Gibco, Life Technologies, Carlsbad, CA), following the manufacturer’s instructions. The RNA quantity and quality were assessed through NanoDrop® ND-1000 Spectrophotometer. RT was performing on 500 ng of total RNA using the High Capacity cDNA Reverse Transcription Kit (Applied Biosystems) according manufacturer’s protocol. TaqMan assays (Applied Biosystems) were used to detect and quantify the following genes: *CDKN1B* (Hs01597588_m1), *CDKN1A* (Hs00355782_m1), *LIG1* (Hs01553527_m1), *PARP1* (Hs00911376_g1), *RAD51* (Hs00153418_m1), TERF2 (Hs00194619_m1), *GAPDH* (Hs02786624 g1). TERRA transcript levels were evaluated from six chromosomes (2q, 7p, 10q, 15q, XpYp, XqYq) amplifying cDNA with Power SYBR™ Green PCR Master Mix (Thermo Fisher Scientific Inc.) and primers reported in Table S2.

### Real-Time quantitative Telomeric Repeat Amplification Protocol (q-TRAP)

We performed real-time qTRAP according the protocol described in Herbert et al. Briefly, we used as positive control (standard) containing known telomerase activity protein lysate from human MCF-7 breast carcinoma cell lines. We created a 1:5 dilution series of standard to yield at least five points. Each sample, including standard dilution series, were analyzed in triplicated. We prepared master mix containing SYBR Green (GoTaq, Promega), 100 ng TS primer per sample, 100 ng ACX primer per sample, 1 mM EGTA and enough RNase free water to bring the final volume of 20 µl. Plate was incubated 30 min at 30 °C in the dark for extension of the substrate by telomerase and then PCR was carried out in a ViiA7 machine (Applied Biosystem) using the following program: 95 °C for 10 min; 40 cycles at 95 °C for 15 s and at 60 °C for 60 s. The Ct values (± standard deviation, SD) of the standard control (MCF-7) were plotted against log[protein] to calculate the linear equation. The Y-intercept and the slope values from the equation are used to quantify the relative telomerase activity (RTA) of samples. For q-TRAP quantification, the RTA of a sample was calculated based on the equation obtained from standard curve y= -2.529x + 21.38 as follows: RTA = 10^[(Ct sample x Yint)/slope]. Sample results were plotted as percentage to control.

### Chromatin ImmunoPrecipitation (ChIP)

ChIP was performed using the Pierce™ Agarose ChIP Kit (Thermo Fisher Scientific, Inc.). Briefly, 1.5 × 10^7^ cells were crosslinked in 1% formaldehyde, lysed and enzymatic digestion with Micrococcal nuclease (MNase). Chromatin was divided into equal amounts of immunoprecipitation with the TRF2 antibody (Abcam, ab13579), or rabbit IgG as negative control (Santa Cruz Biotechnology). Chromatin extracts were incubated on a rotator with 20 µl of ChIP Grade Protein A/G Plus Agarose for 3 h at 4 °C. Bound agarose beads were harvested by centrifugation (12.000 rpm, 15 s) and washed; the precipitated protein-DNA complexes were eluted from washed beads and incubated twice at 65 °C for 1.5 h with NaCl and Proteinase K to revert cross-links. Purified DNA was subjected to qPCR using Power SYBR™ Green PCR Master Mix (Thermo Fisher Scientific Inc.) and telomere primers (Table S2) as previously described [22]. Results are expressed as fold change as compared to IgG set as 1.

### Microarray Gene Expression Profiling (GEP)

GEP was performed on NCI-H929 cells after 24 or 48 h of exposure with 2.5 µM or 5 µM *hit* 17, or DMSO as control. Total RNA was extracted by RNeasy Mini kit (Qiagen, Hilden, Germany). A total of 100 ng RNA was processed using the GeneChip® WT PLUS Reagent Kit (Applied Biosystems) and hybridized to GeneChip® Clariom D human array (Applied Biosystems) according to manufacture standard procedures. Arrays were washed and stained using the GeneChip™ Fluidics Station 450 and scanned using the GeneChip™ Scanner 3000. CEL files were analyzed by Transcriptome Analysis Console v4.0 (Applied Biosystems). Differential expression analysis was assessed using the Gene Level-SST-RMA Summarization Method, a fold change (FC) ≤ -1.5 or ≥ 1.5 and a *p*-value ≤ 0.05. Annotation were performed by the Clariom_D_Human.na36.hg38.transcript.csv. Functional characterization of TERRA was performed using the gene set enrichment analysis (GSEA) software version 4.0.2, a tool to enrich the target pathways with statistically significant differences between *hit* 17 treated *versus* untreated cells. The gene set files selected from Molecular Signatures Database (MSigDB) and used for this analysis was “hallmark gene sets” browse 50 gene sets that represent specific well-defined biological states or processes. Gene set results were considered significant if the false discovery rate was ≤ 0.25 in a number of 1000 permutations.

### Mass spectrometry

We employed mass spectrometry to assess direct binding to the TERT promoter by *hit* 17, employing a previously reported method [23]. Briefly, the oligonucleotide 5’-GGGGAGGGGCTGGGAGGG-3’, corresponding to the hTERT promoter G4 [24], was heat denatured in MS buffer (HFIP 120 mM/TEA pH 7.4, KCl 0.8 mM, Isopropanol 20%) for 5 min at 95 °C, and gradually cooled to room temperature to allow the correct folding. After 4 h, *hit* 17 was added both at 2X and 4X the concentration of oligonucleotide, and samples were incubated overnight at room temperature. Samples were subsequently analyzed by direct infusion electrospray ionization (ESI) on a Xevo G2-XS QTOF mass spectrometer.

### Validation of gene expression pattern

Three independent Affymetrix GeneChip™ Human Genome U133 Plus 2.0 datasets were used to validate the biological value of the DNA repair signature. The GSE5900 contains expression data from plasma cells of 22 healthy donors (HD). The GSE19784 includes 320 newly diagnosed MM patients. From GSE19784 we randomly selected a training cohort and three validation cohorts, namely MM_1_ and MM_2_, MM_3_, MM_4_, respectively The ratio of MM samples included in the training and validation cohorts was approximately 1:1. CEL files from GSE5900 and GSE19784 were downloaded from NCBI Gene Expression Omnibus. All microarray data were normalized by the robust multichip average (RMA) algorithm with the default option using TAC software. We applied a FC ≤ -1.5 or ≥ 1.5 and a *p*-value ≤ 0.05. GSE19784 sample groups, included and analyzed in this study, are listed in Table S3. The GSE4581 dataset contains 414 untreated MM patients and was used to validate the prognostic signature. Survival analyses were performed with the tool-web GenomicScape (http://www.genomicscape.com).

### Animals and in vivo model of human MM

Male CB-17 NOD.SCID mice (6- to 8-weeks old; Harlan Laboratories, Inc., Indianapolis) were housed and monitored at University Magna Graecia Animal Research Facility. The rational design, the use of mice and the experimental procedures were approved by the National Directorate of Veterinary Services, Italy (n. 1138/2020-PR). Two independent animal experiments were conducted. In the first mice were subcutaneously inoculated into the interscapular area with 5 × 10^6^ NCI-H929 cells while the second was performed by the use of 5 × 10^6^ AMO-1 cells. When tumors became detectable by palpation (100–200 mm^3^, approximately 10 days after the injection of MM cells) mice were randomized into 2 groups (5 animals per group) and treated intraperitoneally with 4 mg/kg of *hit* 17 or saline solution in control group. Tumor sizes were measured as previously described [25, 26]. The investigator was blinded to group allocation of each animal.

### Histology and immunohistochemistry (IHC)

Retrieved tumors from animals were fixed in 4% buffered formaldehyde then, after least 24 h, washed, dehydrated, and embedded in paraffin. Four-µm thick sections were mounted on poly-L-lysine slides and stained with hematoxylin–eosin (H&E). For light microscopy analysis by an optical microscope AXIO Scope.A1 (Zeiss Oberkochen, Germany). For IHC, 3-µm-thick tumor slices were deparaffinized and treated with Epitope Retrieval Solution 2 (EDTA-buffer pH 8.8) at 98 °C for 20 min. After washing, peroxidase was blocked by exposing samples to the Bond Polymer for 10 min. All procedures were performed with the Benchmark XT-Automated Immunohistochemistry instrument (Ventana Medical Systems, Oro Valley, AZ, USA). Tissues were washed again and then incubated with the rabbit monoclonal primary antibody CONFIRM^TM^anti-Ki-67 (Ventana, clone 30–9; 1∶150). Tissues were then incubated with DAB-Chromogen (8 min) and slides were counterstained with hematoxylin (12 min).

### Statistical analysis

Each experiment was performed at least three times and values are reported as means ± standard deviations. Comparisons between groups were made with the Student t-test, while statistical significance of differences among multiple groups was determined by GraphPad software (www.graphpad.com). Graphs were obtained using GraphPad Prism version 6.0. *P*-values less than 0.05 were accepted as statistically significant.

## Results

### *Hit* 17 suppresses proliferation and induces apoptosis in MM cells

**Fig. 1:**
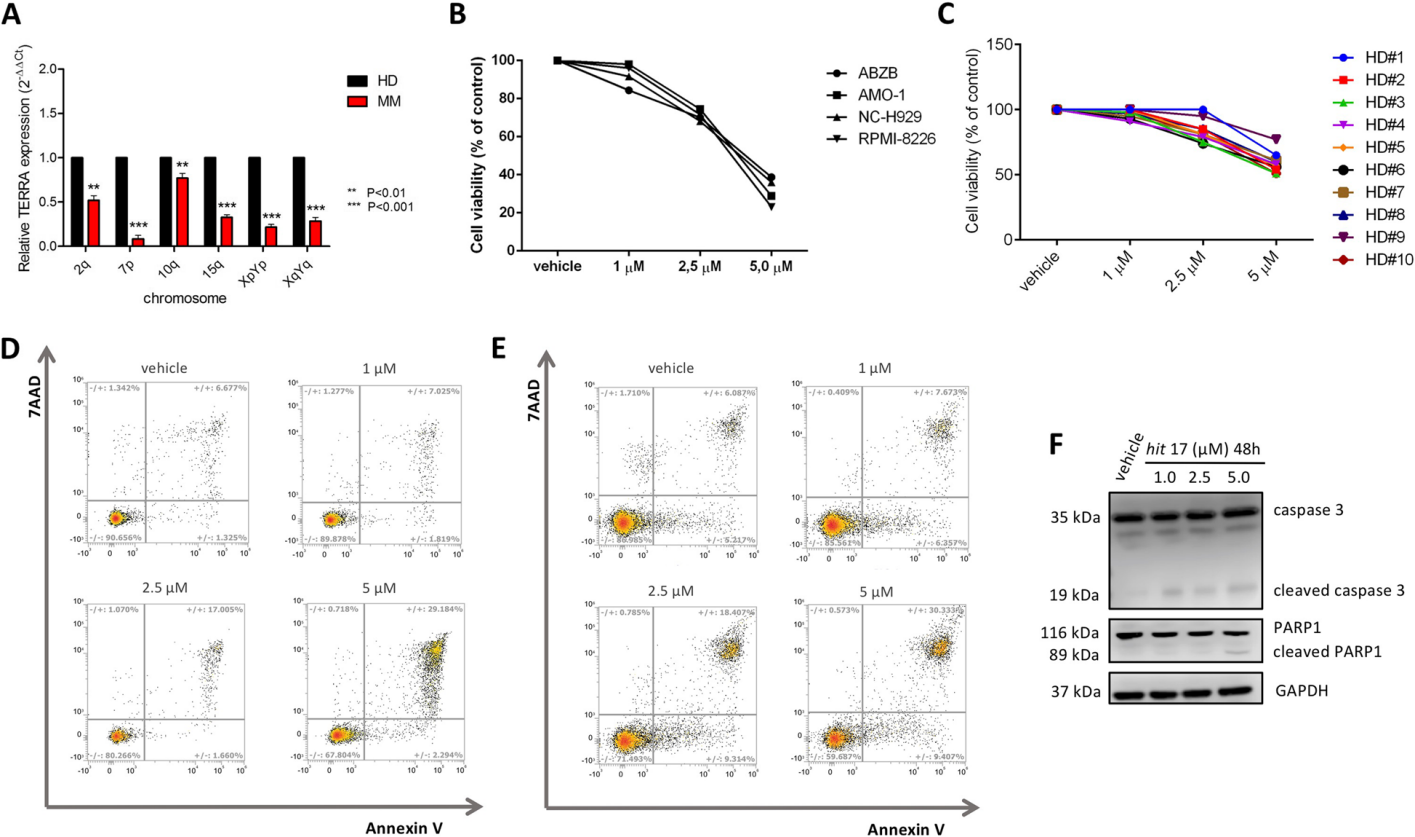
*Hit* 17 inhibits MM cell viability and activates apoptotic signaling. **A** RT-qPCR of TERRA in MM patients and HD. **B** Four MM cell lines and (**C**) PBMCs from 10 healthy donors were treated with *hit* 17 at indicated concentrations for 48 h, followed by Cell Titer-Glo assay. NCI-H929 (**D**) and AMO-1 (**E**) cells were treated with *hit* 17 (1, 2.5, and 5 μM) for 48 h. Cells were then stained with Annexin V-PE and 7-AAD, followed by flow cytometry analysis. The number of each quadrant was presented in %. **F** WB analysis of cleaved caspase-3 and cleaved PARP1 in the NCI-H929 cell line. Results shown in **B**, **D**-**F** are the average of three independent biological replicates. Error bars are SD

We first explored the role of TERRA in MM at mRNA level. Due to the absence of TERRA expression in public data repositories, we performed RT-qPCR in a pilot cohort of patients including four MM patients and four HD. We used six different couple of primers, each specifically designed to detect TERRA transcripts from six different chromosome regions (2q, 7p, 10q, 15q, XpYp and XqYq). We observed a decreased expression of TERRA in plasma cells from MM as compared to PBMCs from normal donors (Fig. 1A). We further evaluated TERRA basal levels in NCI-H929 and AMO-1 cell lines with respect to three ALT cell lines (SAOS-2, SKOV-3, and RMG1), that usually display high levels of TERRA. TERRA levels were significantly lower in MM compared with ALT with a fold change expression (MM vs ALT) calculated by 2^^−∆∆Ct^ method in the range of 0.032—0.260 (Fig. S1A). The biological effect of TERRA G4 stabilization mediated by *hit* 17 was evaluated on MM cell lines and ALT cell lines as positive control. A panel of four MM cell lines, including bortezomib-resistant ABZB cells, were cultured for 48 h at different concentrations of *hit* 17 (1 µM, 2.5 µM, 5 µM). We observed that *hit* 17 exerted an anti-proliferative effect in all MM cell lines, showing IC_50_ (half-maximal inhibitory concentration) values ranging from 2.5 to 5 µM at 48 h (Fig. 1B). ALT cell lines required a concentration of 20–40 µM to reach the IC_50_ after 48 h of *hit* 17 treatment (Fig. S1B). Moreover, to investigate the sensitivity of normal cells to *hit* 17, we treated at the same conditions a number of ten PBMCs samples from HD. Cell viability assay on treated normal cells did not reveal any cytotoxic effect in the same range of IC_50_, suggesting an MM-specific vulnerability by this compound (Fig. 1C). To delineate the mechanism of cell death induced by *hit* 17 in MM cells, NCI-H929 and AMO-1 cells were treated with different concentrations of *hit* 17 and apoptosis was evaluated. As shown in Fig. 1D and 1E, *hit* 17 induced apoptosis in both cell lines with a dose-dependent increase of around 4-fold at 5 µM treatment respect to the control. This alteration was confirmed by WB analysis showing an increase of cleaved caspase-3 and cleaved PARP1 in NCI-H929 cells (Fig. 1F).

### TERRA G4 stabilization by *hit* 17 enhances TERRA level

**Fig. 2:**
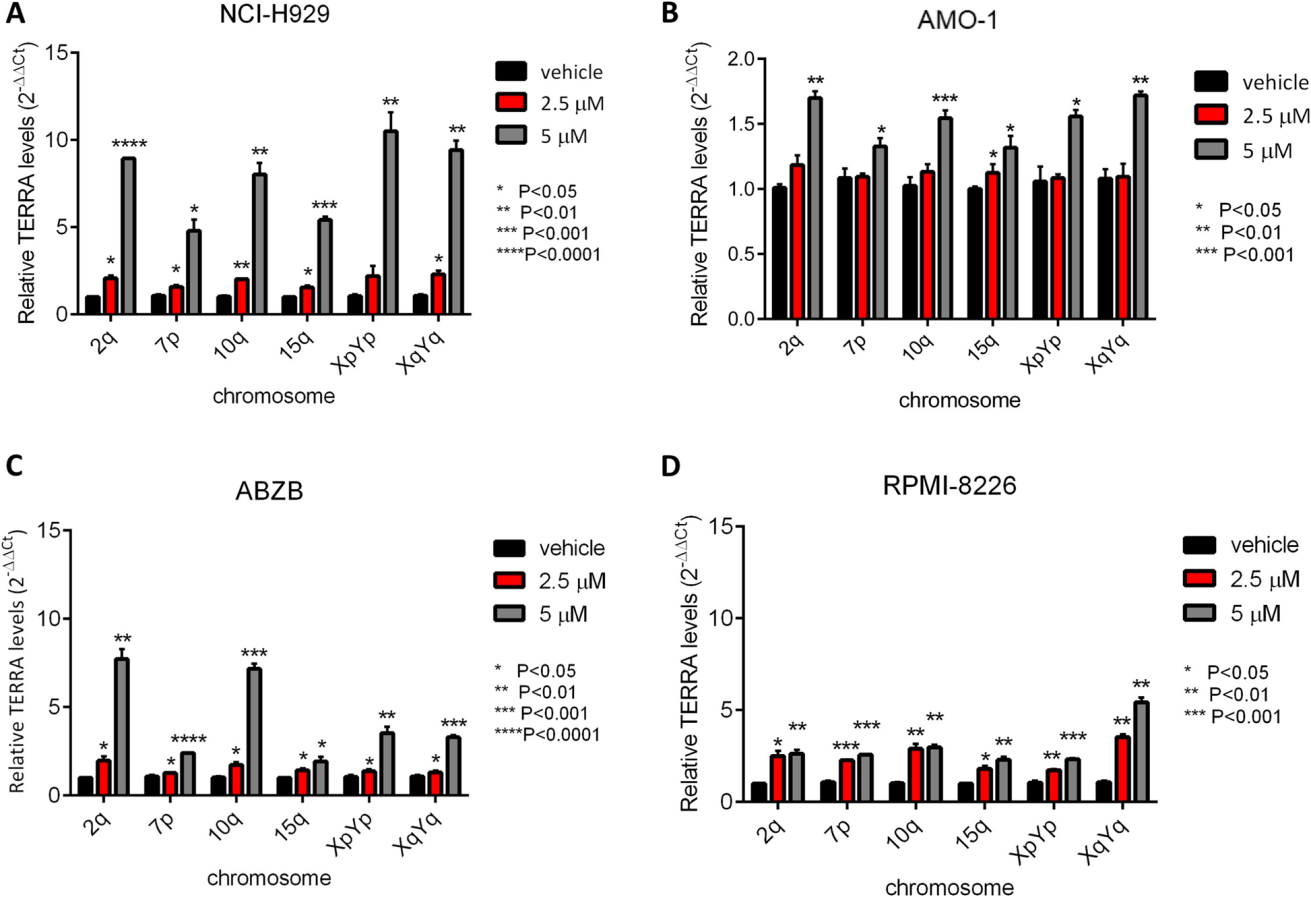
TERRA expression levels increase after *hit* 17 treatments in MM cell lines. Representative RT-qPCR of TERRAs after 48 h of *hit* 17 treatments in NCI-H929 (**A**) AMO-1 (**B**), ABZB (**C**), and RPMI-8226 **D**. Results shown are the average of three independent biological replicates and error bars are SD

To analyze differences in TERRA expression following treatment, we performed RT-qPCR in MM cell lines, ALT cell lines and HD. We observed that TERRA levels increased after *hit* 17 treatments in a dose-dependent manner with remarkable fold change at the dose of 5 µM ranging from 2- to 10-fold in MM cell lines (Fig. 2A-D) and at the dose 20 µM in the range of 2- to 20-fold in ALT cell lines (Fig. S2A-C). In contrast, in normal cells we did not appreciate a significant up-regulation of TERRA after *hit* 17 treatments (Fig. S2D).

### *Hit* 17 modulates TERRA downstream targets

**Fig. 3:**
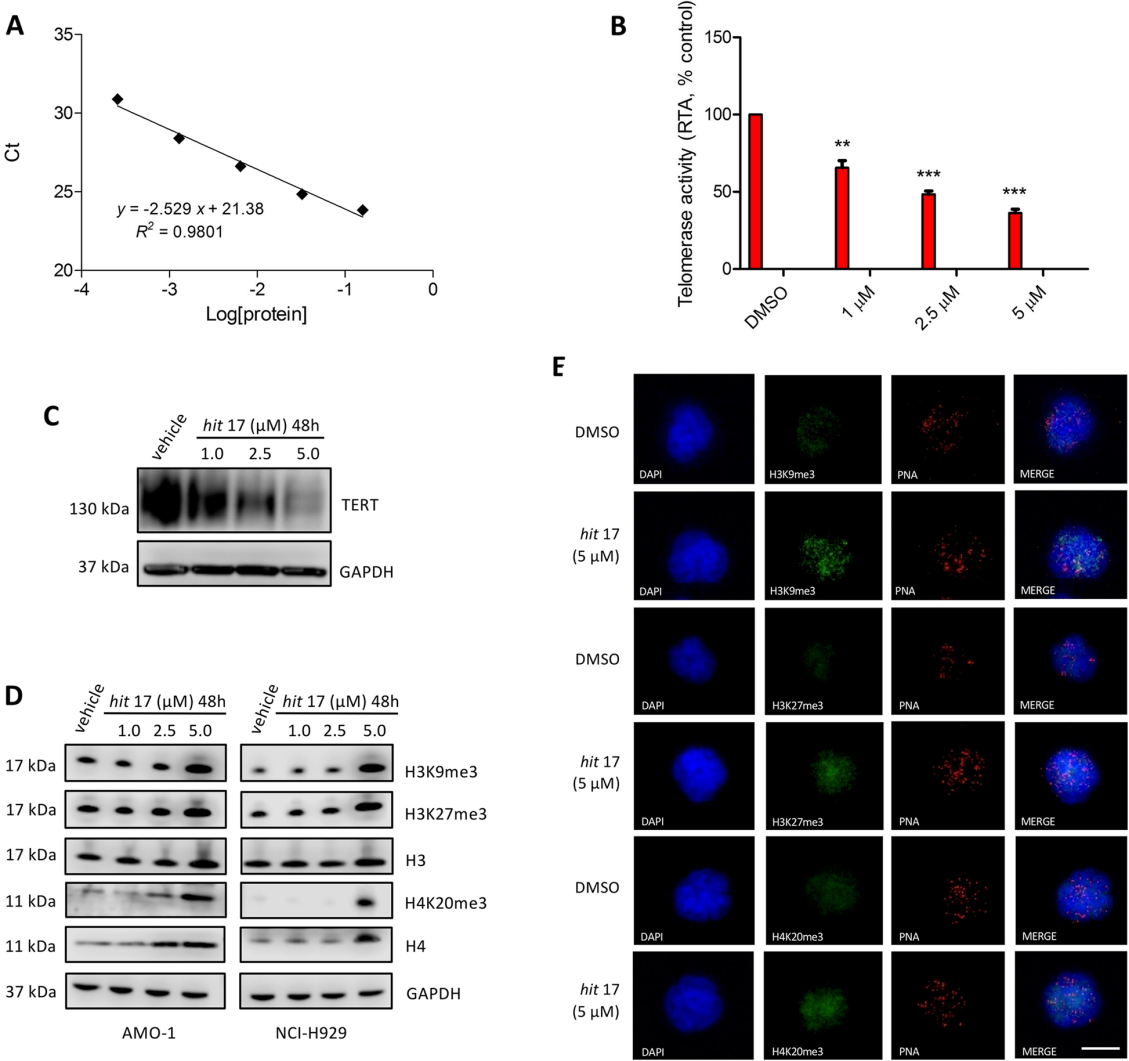
**A** Standard curve and linear relationship for q-TRAP. The Ct values (± s.d.) of the standard control (MCF-7) were plotted against log [protein] to calculate the linear equation. The Y-intercept and the slope values from the equation are used to quantify the RTA of samples. **B** Telomerase activity as measured by q-TRAP assay. For q-TRAP quantification, the RTA for a sample was calculated based on the equation obtained from the standard curve y = -2.529 x + 21.38; RTA = 10^ [(Ct sample x Yint)/slope]. Sample results are plotted as percentage to control. **C** Representative WB analysis of TERT in NCI-H929 cell lines 48 h after *hit* 17 treatments. **D** Representative WB analysis of histone H3, H4 and try-methylation of H3 at lysine 9 (H3K9me3), H3 at lysine 27 (H3K27me3) and H4 at lysine 20 (H4K20me3) in NCI-H929 cell lines 48 h after *hit* 17 treatments. **E** Representative images showing detection of heterochromatin trimethylation markers by immuno-FISH in *hit* 17 and control (DMSO) in NCI-H929 cell lines. Interphase cells were stained with anti-H3K9me3 (green), anti-H3K27me3 (green), anti-H4k20me3 (green) and telomeric Cy3-labeled probe (red). Nuclei were counterstained with DAPI (blue). Scale bar, 10 μm. ** *P* <0.01; *** *P* <0.001

TERRA transcripts repress telomerase activity and maintain a heterochromatic state at the ends of chromosomes through the interaction with PRC2. TERRA-PRC2 complex promotes deposition of H3K9me3, H4K20me3, H3K27me3 and HP1 protein to chromosome ends. To assess whether *hit* 17 modulates downstream targets of TERRA G4, we performed q-TRAP for the detection of telomerase activity and WB to evaluate the expression level of human telomerase reverse transcriptase (hTERT), and the trimethylation of histone H3 (lysine 9 and 27) and histone H4 (lysine 20). We found that NCI-H929 treated with *hit* 17 showed a decrease of telomerase activity (Fig. 3A, B) combined with the reduction of hTERT protein (Fig. 3C), while all three histone trimethylation marks resulted increased in both NCI-H929 and AMO-1 cell lines (Fig. 3D). Immunofluorescence combined with a Cy3-labeled (CCCTAA)_3_ probe demonstrated a preferential deposition of heterochromatin trimethylation markers at telomeres as reported in Fig. 3E. These results demonstrate that *hit* 17 is a potent inhibitor of telomerase in vitro and that the amount of telomeric heterochromatin markers such as H3K9me3, H4K20me3 and H3K27me3 depends on the level of TERRA transcripts.

To exclude a direct *hit* 17 binding to DNA G4 in the promoter of TERT, we employed mass spectrometry and found that the binding percentage of *hit* 17 to the TERT promoter was 12.5 ± 2.2% indicating that *hit* 17 binding to the TERT promoter marginally contributes to inhibition of TERT expression (Fig. S3 A, B).

### *Hit* 17 induces dissociation of TRF2 from telomeres

**Fig. 4:**
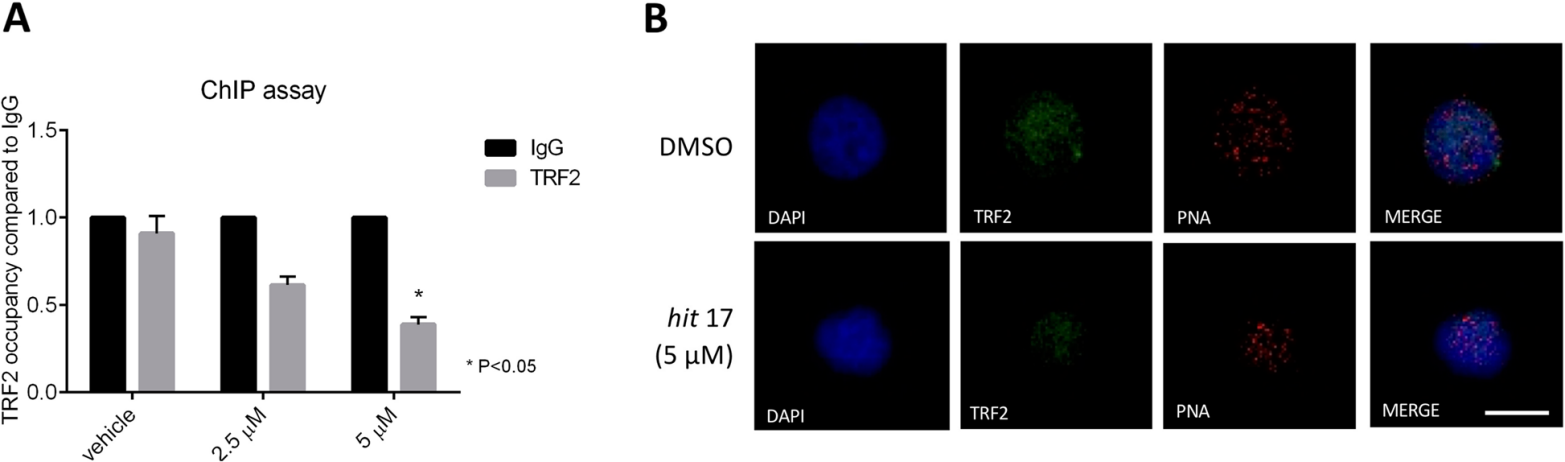
TERRA G4 stabilization disrupts TRF2 binding and leading to strong modulation of its targets. **A** Quantitative PCR of telomeres performed after TRF2 ChIP on NCI-H929 cell line. Results are expressed as fold change respect to IgG set as 1. **B** Representative images showing detection of TRF2 by immuno-FISH in *hit* 17 and control (DMSO) in NCI-H929 cell line. Interphase cells were stained with anti-TRF2 (green) and telomeric Cy3-labeled probe (red). Nuclei were counterstained with DAPI (blue). Scale bar, 10 μm. *P <0.05

TERRA is known to interact with TRF2 increasing its specific binding to telomeric DNA [8]. To understand the effect of *hit* 17 on TRF2 recruitment on telomeres, we performed ChIP on the NCI-H929 cell line after small molecule treatment at 48 h of 2.5 and 5 µM. As shown in Fig. 4A, we observed a dose-dependent TRF2 dissociation of 2-fold change from telomeres, consistent with previously reported observations [27]. We confirmed by immuno-FISH TRF2 displacement from telomeres (Fig. 4B). No modulation of TRF2 expression levels was observed after *hit* 17 treatments (Fig. S4). These results are in line with previous data reporting that TRF2 directly suppresses TERRA transcription through its homodimerization domain. When TRF2 is removed, the higher-order telomere structures are disrupted and the telomeric chromatin becomes accessible to RNAPII for TERRA transcription [28].

### ATM activation by *hit* 17 induces DNA damage and cell cycle inhibition

**Fig. 5:**
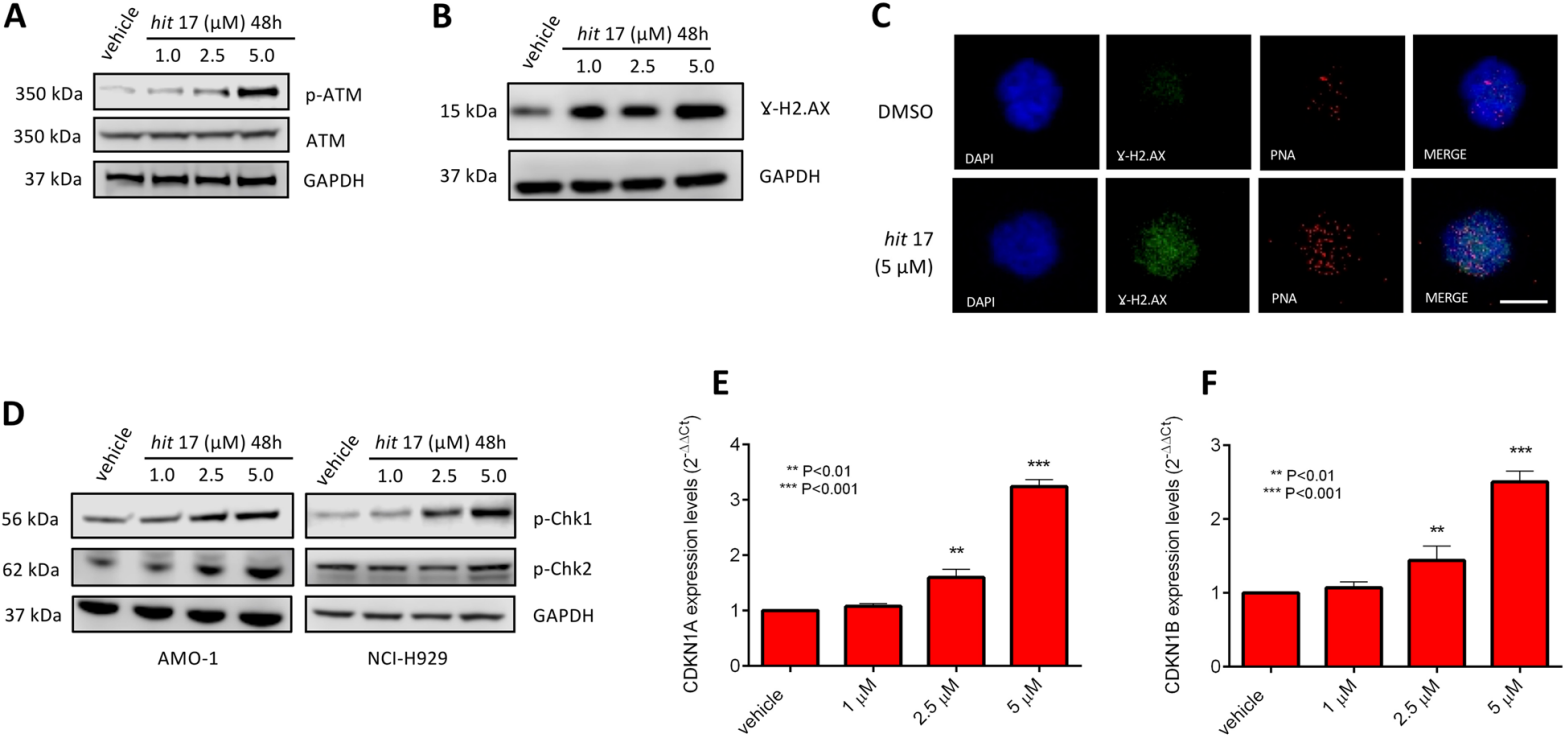
ATM pathway activation after *hit* 17 treatments in MM cell lines. Representative WB analysis of p-ATM (**A**) and γH2AX (**B**) in NCI-H929. **C** Representative images showing detection of γ-H2AX by immuno-FISH in *hit* 17 and control (DMSO) NCI-H929 cell lines. Interphase cells were stained with antiphospho-histone H2AX (green) and telomeric Cy3-labeled probe (red). Nuclei were counterstained with DAPI (blue). Scale bar, 10 μm. **D** WB analysis of p-CHEK1 and p-CHEK2 in both NCI-H929 and AMO-1 cells. Representative expression of *CDKN1A* (**E**) and *CDKN1B* (**F**) in NCI-H929 cell lines by RT-qPCR. **P <0.01; ***P<0.001

Activation of ATM also takes place in response to telomere damage. When telomeres become uncapped due to inhibition of TRF2 binding, phosphorylated ATM associates with telomeres [29, 30] and actives DNA damage response. To assess the possible changes induced by *hit* 17 treatments in this signaling pathway, we analyzed the expression levels of ATM by WB along with its downstream targets, including the cell cycle inhibitors cyclin-dependent kinase inhibitor 1A (CDKN1A, also known as p21) and cyclin-dependent kinase inhibitor 1B (CDKN1B, p27). As reported in Fig. 5, *hit* 17 treatments induced a dose-dependent increase of phosphorylated ATM (p-ATM), CHEK1 (p-Chk1), CHEK2 (p-Chk2) and H2AX (γH2AX) suggesting the activation of DNA damage response pathway in MM cell lines. Consistent with this, there was an accumulation of TIFs containing γH2AX in NCI-H929 cell line treated with *hit* 17 (Fig. 5C). In addition, we observed by RT-qPCR a stronger up-regulation of p21 and p27 in the NCI-H929 cell line at 5 µM of *hit* 17 (Fig. 5E-F). Overall, our data are consistent with previous findings of the TRF2-dependent and ATM-mediated DNA damage response at telomeric ends [31, 32].

### TERRA G4 downstream pathways modulated by *hit* 17

**Fig. 6:**
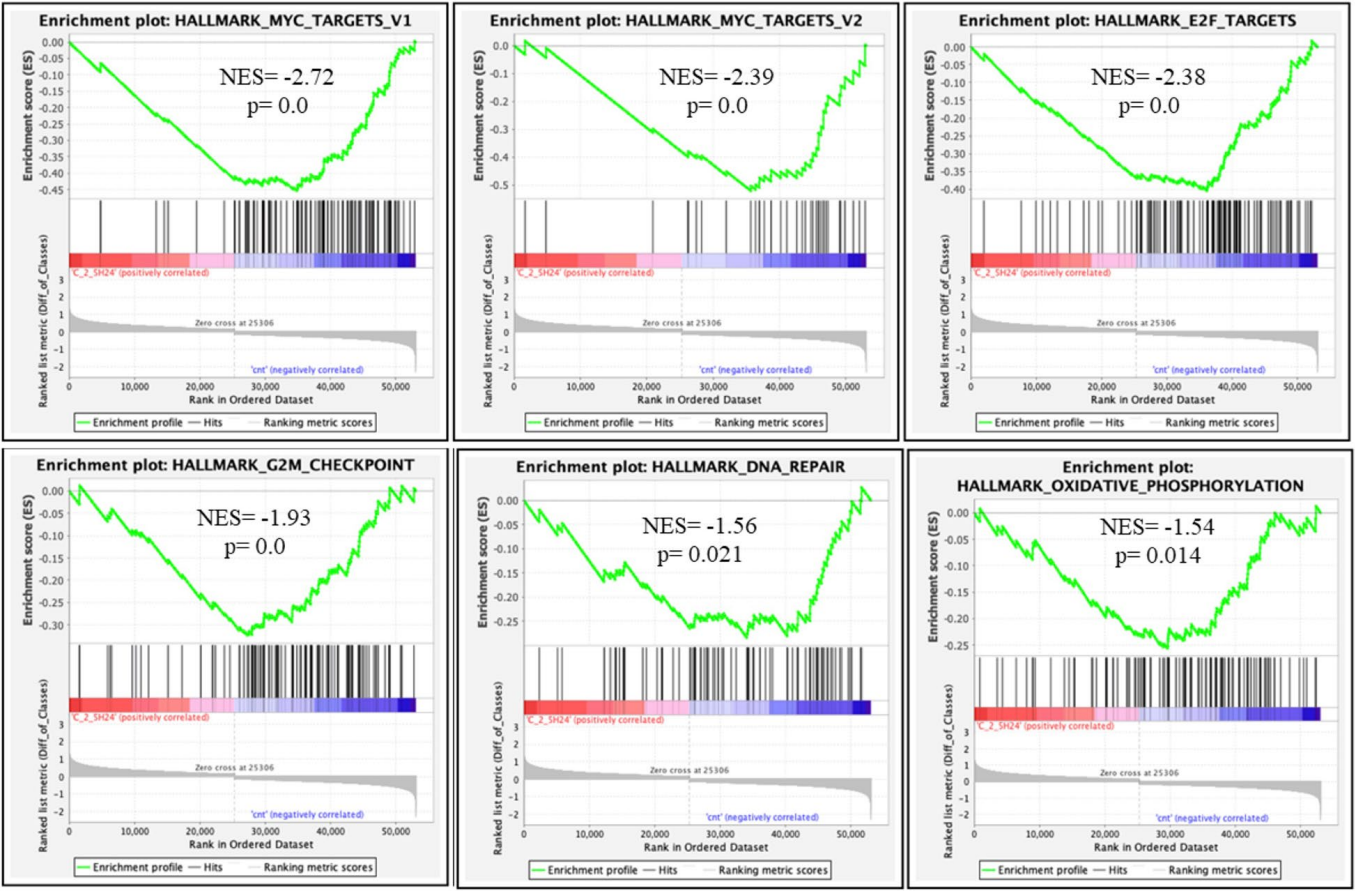
GSEA performed after *hit* 17 treatments on the NCI-H929 cells treated with 2.5 µM of *hit* 17 for 24 h. Plots of significantly enriched HALLMARK gene sets: MYC_TARGETS_V1; MYC_TARGETS_V2; E2F_TARGETS; G2M_CHECKPOINT; DNA_REPAIR; OXIDATIVE_PHOSPHORYLATION. Normalized enrichment score (NES) and the corresponding *p*-value are reported within each graph. A *p*-value of zero (0.0) indicates an actual *p*-value of less than 0.001 (1/1000 permutations)

**Fig. 7:**
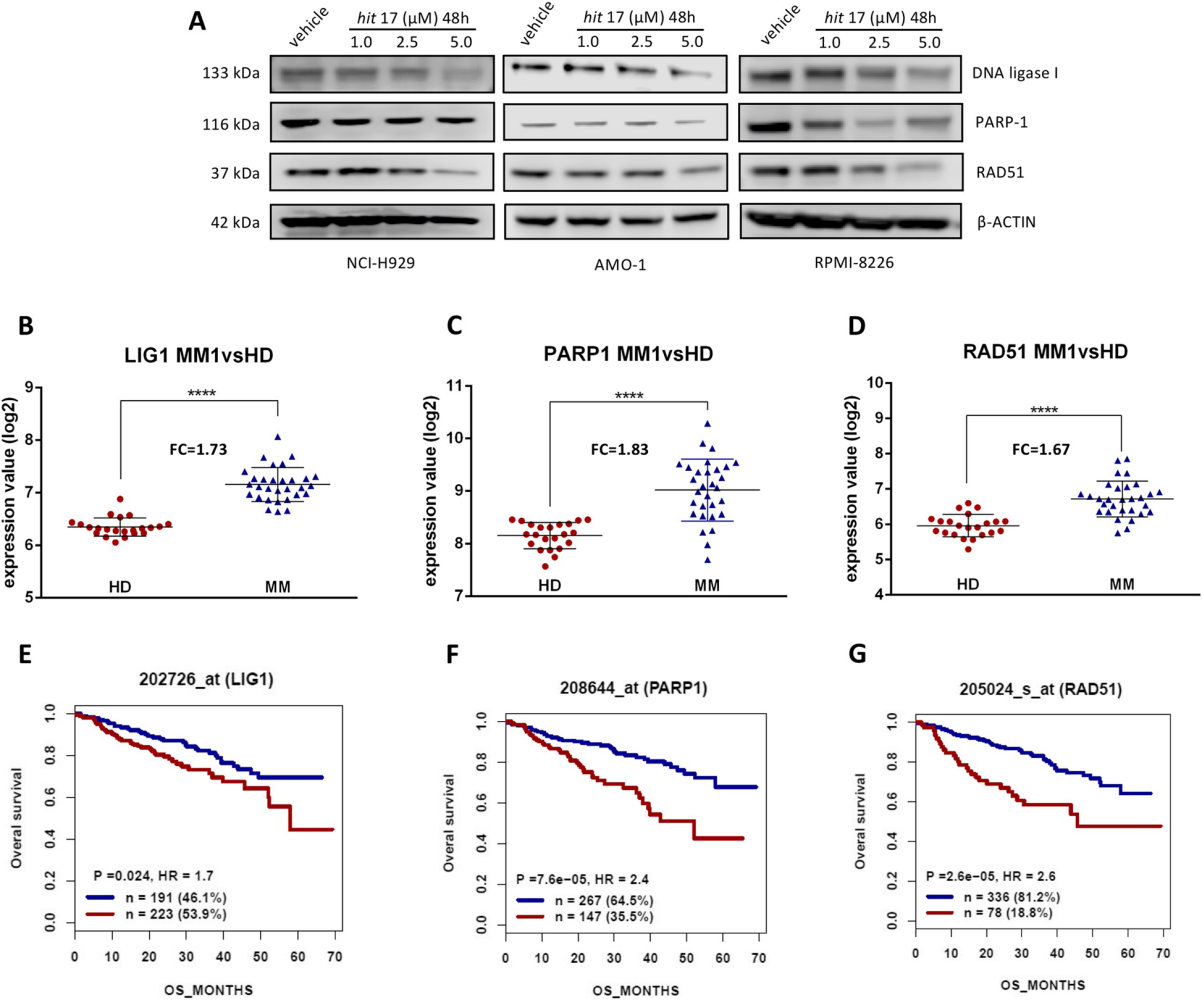
In vitro down-regulation of DNA repair gene signature. **A** WB analysis of DNA ligase I, PARP1 and RAD51 after 48 h of *hit* 17 treatments in the NCI-H929, AMO-1, and RPMI-8226 cell lines. **B**-**D** Gene expression levels of *LIG1*, *PARP1* and *RAD51* in MM samples compared to HD. **E**–**G** The survival analysis of 414 MM patients from GSE4581 classified according to gene expression levels: high (red) or low (blue). We used as a scale to plot OS the days’s scale. FC: fold change; P: p-value; HR: Hazard Ratio; n: number of patients. ****P < 0.0001

To explore molecular mechanisms induced by *hit* 17 in MM, we performed GEP on NCI-H929 cell line at two different doses (2.5 µM and 5 µM) and times (24 and 48 h) of treatment. Microarray profiles were analyzed by GSEA. This analysis revealed a significant enrichment of 31 gene sets (Table S1) after 48 h of *hit* 17 treatment at 5 µM. In particular, TNFα, p53, and hypoxia pathways were significantly up-regulated in *hit* 17-treated cells with a normalized enrichment score (NES) ≥ 2 and *p* < 0.001. Conversely, targets of E2F and MYC, cell cycle, oxidative phosphorylation, mitotic spindle and DNA repair were significantly inhibited in NCI-H929-treated cells (NES ≤ -3, *p* < 0.001). The modulation of these pathways was already observed after 24 h of exposure and 2.5 µM concentration, suggesting that these perturbations are early events in *hit* 17 treatment (Fig. 6). Focusing on the DNA repair pathway, and in particular on key regulators such as PARP1, RAD51, and DNA ligase I, we validated the down-regulation of this gene signature by RT-qPCR, confirming the inhibition of the DNA repair process (Fig. S5). Accordingly, the down-regulation of this DNA repair gene signature was also validated in three MM cell lines after *hit* 17 treatments (Fig. 7A). The expression level of major oncogenes harboring G4 forming sequences were evaluated. In particular, we focused on the expression level of oncogenes like *BCL2*, *KRAS* and *MYC*. Among these, we found a significant down-regulation of MYC thus we cannot exclude that *hit* 17 at higher doses could bind and stabilize G4 located at *MYC* promoters.

### Diagnostic and prognostic evaluation of DNA repair gene signature

The gene expression patterns of DNA repair gene signature were further validated using GSE5900 and GSE19784 cohorts. *LIG1*, *PARP1*, and *RAD51* expression are remarkably higher in MM samples when compared with HD samples (all *P* < 0.0001; Fig. 7B-D and Fig. S6). Survival analyses were performed in the GSE4581 dataset. As revealed in the Fig. 7E-G, MM patients with high expression of these three genes demonstrated a significantly unfavorable overall survival (OS) than patients with low expression (*LIG1*: probe-set 202726_at, HR = 1.7, P = 0.024; *PARP1*: probeset 208644_at, HR = 7.6 × 10^–5^; *RAD51*: probe-set 205024_s_at, HR = 2.6, P = 2.6 × 10^–5^). In summary, the DNA repair gene signature identified in this study show a correlation with MM outcome.

### In vivo activity of *hit* 17

**Fig. 8:**
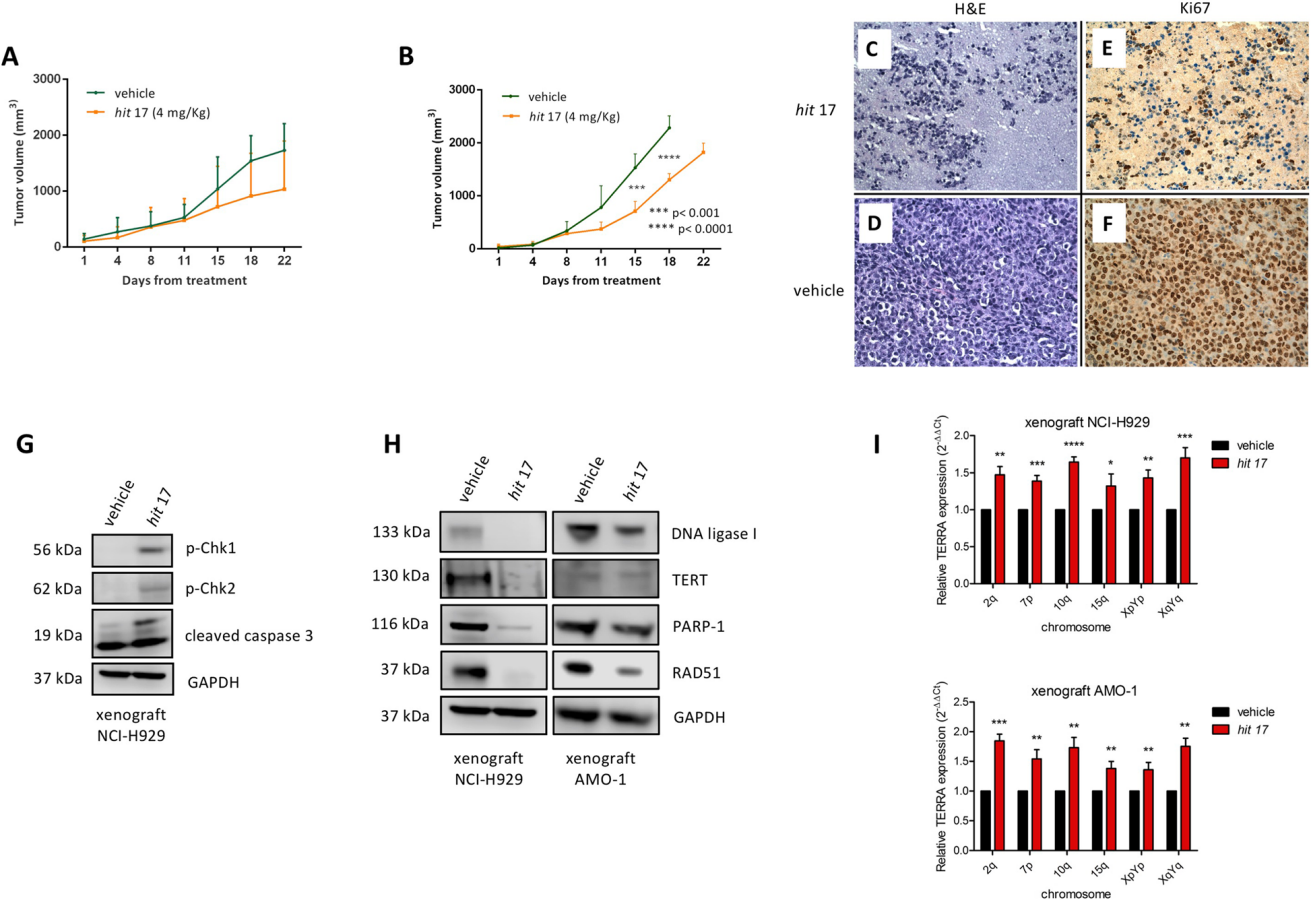
In vivo activity of *hit* 17 in MM xenograft models. In vivo growth of NCI-H929 and AMO-1 xenograft intraperitoneally treated with *hit* 17 at dose of 4 mg/kg. Average tumor volume of NCI-H929 (**A**) and AMO-1 xenograft (**B**) ± SD (5 animals per group) is shown, *p > 0.05. H&E staining (**C** and **D**, 40x) and IHC evaluation of Ki67 (**E** and **F**, 40x) of retrieved tumors from NCI-H929 xenograft after *hit* 17 treatments. **G** WB analysis of p-CHEK1, p-CHEK2, cleaved-caspase-3 in lysates from a representative NCI-H929 retrieved xenografts. GAPDH was used as control. **H** WB analysis of DNA ligase I, TERT, PARP1, and RAD51 in NCI-H929 and AMO-1 retrieved xenografts. **I** Representative RT-qPCR of TERRAs from three NCI-H929 and AMO-1 xenograft samples after *hit* 17 treatments. *P <0.05; **P<0.01; ***P <0.001; ****P<0.0001

To assess the translational relevance of *hit* 17*,* we evaluated the anti-tumor activity against human MM xenografts in NOD-SCID mice. When NCI-H929 MM xenografts became palpable, mice were randomized into 2 groups and treated with *hit* 17 or saline. Intraperitoneal injection of 4 mg/kg was performed everyday treatment, 5 per week, for three weeks. We observed significant inhibition of tumor growth in *hit* 17 treated animals as compared to the control group (*p* < 0.05) in both experimental settings (Fig. 8A-B). However, standard deviation in both groups indicates a variability among animals. Staining with H&E showed strong tumor shrinkage in the treated xenografts (Fig. 8C-D). Ki-67 in IHC revealed a reduction of the proliferative index from 95% in the control group to 10% in treated group (Fig. 8E-F). Further, WB analysis of retrieved xenografts showed activation of DNA damage markers, the induction of apoptotic process by caspase-3, the down-regulation of TERT, and DNA repair gene signature in treated mice compared to the control group, consistent with *hit* 17 mechanism of action (Fig. 8G-H). The biological effect of *hit* 17 to increase TERRA expression levels was confirmed in three NCI-H929 and AMO-1 xenograft samples as shown in Fig. 8I.

## Discussion

Genomic instability, an increased tendency to acquire genomic alterations, is a cancer hallmark and its frequency varies across different tumors [33]. Given its prognostic value, understanding mechanisms that lead to genomic instability is a crucial issue for the application of precision medicine. MM represents a suitable disease model to investigate genomic instability as it evolves through premalignant stages characterized by progressive accumulation of genomic alterations [34]. Several mechanisms have been proposed as causes of genomic instability in MM [1], including the dysfunction of the DNA repair machinery [35, 36]. Another important source of chromosomal instability is telomere uncapping and recently it has been demonstrated that telomere structure correlates with aggressiveness in MM at diagnosis [37, 38]. On the other hand, in the last years, several studies have highlighted how ncRNAs, among all miRNAs and lncRNAs, are driver regulators in biological processes and molecular functions and their dysregulation is a frequent event in numerous cancers [39, 40], including MM [41–44]. In this scenario, we explored in MM the functional role of TERRA, a class of lncRNA known to be involved in telomere stability. Transcriptional dysregulation of TERRA has been previously reported in several cancers although at different trends of expression. For instance, some authors reported that TERRA levels were lower in the tumor of patients with squamous cell carcinoma of the head and neck and correlated with a worse clinical outcome than patients in which TERRA in the tumor was higher or similar compared to the normal tissue [13]. However, to our knowledge, the role of TERRA in the pathogenesis of MM has not been studies. Therefore, we underwent TERRA investigation by targeting with *hit* 17, a small molecule that can bind and stabilize the G4 conformation. Here, we provide evidence of anti-MM activity of *hit* 17 in vitro and in vivo models*.* Consistently, we observed in vitro inhibition of proliferation, induction of apoptosis, G2 cell cycle arrest and in vivo tumor growth inhibition. Importantly our data show that MM cell lines were more susceptible to treatment with *hit* 17 than normal cells and expressed lower TERRA levels as compared to normal cells. We can hypothesize that the difference in susceptibility to *hit* 17 treatments between normal and MM cells can be partly due to intrinsic characteristics of tumor cells such as the mutational landscape. For instance, MM cell lines harbor alterations in key genes that could enhances the ability of *hit* 17 to induce DNA damage response. We explored the molecular mechanisms underlying this anti-tumor effect in MM cell lines by focusing on TRF2 due to the high binding affinity of this shelterin protein with TERRA G4. Specifically, by ChIP assay, we observed that TERRA G4 stabilization by *hit* 17 reduces TRF2 DNA binding to telomeres, in a dose-dependent manner, with subsequent activation of ATM and its downstream targets. Displacement of TRF2 by *hit* 17 treatment increases TERRA levels from 2-fold to 10-fold at the dose of 5 µM. This result is according to the work of Porro et al. in which the authors demonstrated an up-regulation of TERRA following TRF1 or TRF2 loss but not upon depletion of the other shelterin components [23]. *Hit* 17 was able to increase TERRA activity that was demonstrated through the inhibition of telomerase as well as through the increased activity of TERRA-PRC2 complex. We also found an inverse correlation between TERT and TERRA expression levels after *hit* 17 treatments, being TERRA up-regulated and TERT down-regulated, and in addition, by mass spectroscopy, we excluded direct binding to the TERT promoter by *hit* 17. This trend is in accordance with results from previous published studies [45, 46]. In in vivo experiments, we found that systemically administered *hit* 17 exerted a significant anti-MM activity as demonstrated by tumor shrinkage associated with a strong reduction of proliferative Ki67 index. Interestingly, whole transcriptome analysis revealed a signature of three down-regulated genes (*LIG1*, *PARP1*, and *RAD51*) after *hit* 17 treatment that we confirmed also in MM cells retrieved from xenografts. Moreover, in two datasets of MM patients, we disclosed the association of high *LIG1*, *PARP1* and *RAD51* expression with worse clinical outcome. Different studies have reported overexpression of these genes in cancer including MM. For instance, in a previous study work, we observed that the expression levels of *PARP1* increased during disease progression and in high-risk MM subgroups harboring t(4;14) and t(14;16) translocations [47]. *LIG1*, *PARP1*, and *RAD51* are involved in DNA damage response (DDR) through different DNA repair pathways: non-homologous end joining (*LIG1* and *PARP1*) and homologous recombination (*RAD51*). The DDR has emerged as a hallmark in cancer resulting in the development of DDR inhibitors such as PARP inhibitors. A potential role for PARP inhibitors in the treatment of MM has already been proposed for MM patients with acquired homologous recombination deficiency [48]. In addition, several small molecules and peptides inhibiting RAD51 have been developed to increase MM cancer cell sensitivity to doxorubicin or melphalan [49, 50]. In this context, our results provide the rationale to consider TERRA as a new drug target that could improve the treatment of MM in combination with DNA-damaging agents.

## Conclusions

The relationship between lncRNAs and cancer development and progression is well-recognized, thus the modulation of oncogenic or tumor suppressor lncRNAs remains an unchallenged resource. To our knowledge this is the first report that provides the relevant role of TERRA in MM. Despite some limitations of this study such as the low number of enrolled patients and animals, we believe that this study opens a new strategy for targeting the pathways involved in genomic instability in human cancer such as MM, and provides a new powerful agent against MM.

## Supplementary Information

**Additional file 1: Figure. S1. A** Basal TERRA levels in NCI-H929 and AMO-1 cell lines respect to three ALT cell lines (SAOS-2, SKOV-3 and RMG1) that usually display high levels of TERRA. **B** ALT cell lines were treated with *hit* 17 at 2.5, 5, 10 and 20 μM for 48 hours, followed by Cell Titer-Glo assay.

**Additional file 2: Figure. S2. A-C** RT-qPCR of TERRAs after 48 hours of *hit* 17 treatments on SAOS-2, SKOV-3, and RMG1. **D** RT-qPCR of TERRAs after 48 hours of *hit* 17 treatments on PBMCs from five HD donors. * *P*<0.05; **P<0.01; *** *P*<0.001; **** *P*<0.0001. Results shown in B-D are the average of two independent biological replicates. Error bars are SD.

**Additional file 3: Figure. S3. A**, **B** MS spectra of TERT (blue squares) incubated with *hit* 17 (red circles). Samples (5 μl) containing 5 μM of TERT oligonucleotide and 10 μM (panel A) or 20 μM (panel B) of *hit *17 were incubated in MS buffer (HFIP 120 mM/TEA pH 7.4, KCl 0.8 mM, Isopropanol 20%) overnight before MS analysis.

**Additional file 4: Figure. S4.** RT-qPCR of TERF2 encoding TRF2 protein. *Hit* 17 treatments on the NCI-H929 cell line showed no modulation of the expression level of TRF2.

**Additional file 5: Figure. S5. **Validation of DNA damage gene signature. RT-qPCR of selected genes (*LIG1*, *PARP1*, *RAD51*) involved in DNA damage response in the NCI-H929 cell line after *hit* 17 treatments.

**Additional file 6: Figure. S6. **DNA repair gene signature validation. Gene expression levels of *LIG1*, *PARP1* and *RAD51* in MM2, MM3, and MM4 validation cohorts from GSE19784 each compared to HD samples from GSE5900. FC= fold change. ****P<0.0001

**Additional file 7: Table S1.** List of significantly enriched gene sets performed on NCI-H929 with GSEA software.

**Additional file 8: Table S2.** Primer sequences.

**Additional file 9: Table S3. **Samples from GSE19784 included and analyzed in this study.

## Data Availability

The datasets used and analyzed in this study are available from the corresponding author upon reasonable request.

## Abbreviations

7-AAD: 7-Aminoactinomycin D
ChIP: Chromatin immunoprecipitation
DMSO: Dimethyl sulfoxide
ESI: Electrospray ionization
FBS: Fetal bovine serum
FC: Fold change
GAR: Glycine-arginine rich
G4: G-quadruplex
GEP: Gene expression profiling
GSEA: Gene set enrichment analysis
HD: Healthy donors
H&E: Hematoxylin & eosin
hTERT: Human telomerase reverse transcriptase
IC_50_: Half-maximal inhibitory concentration
IF: Immunofluorescence
IHC: Immunohistochemistry
lncRNAs: Long non-coding RNAs
MGUS: Monoclonal gammopathy of undetermined significance
MM: Multiple myeloma
MNase: Micrococcal nuclease
MSigDB: Molecular signatures database
PBMCs: Peripheral blood mononuclear cells
PRC2: Polycomb repressive complex 2
q-TRAP: Quantitative telomeric repeat amplification protocol
RMA: Robust multichip average
RNAPII: RNA polymerase II
RTA: Relative telomerase activity
RT-qPCR: Reverse transcription quantitative PCR
SMM: Smoldering multiple myeloma
TRF2: Telomeric repeat‐binding factor 2
TIF: Telomere dysfunction-induced foci
TERRAs: Telomeric repeat-containing RNAs
WB: Western blotting
