# Supplementary figures and images for: TERRA G-quadruplex stabilization as a new therapeutic strategy for multiple myeloma
Source: J Exp Clin Cancer Res. 2023 Mar 27;42:71. doi: 10.1186/s13046-023-02633-0 (PMC10041726; doi:10.1186/s13046-023-02633-0)

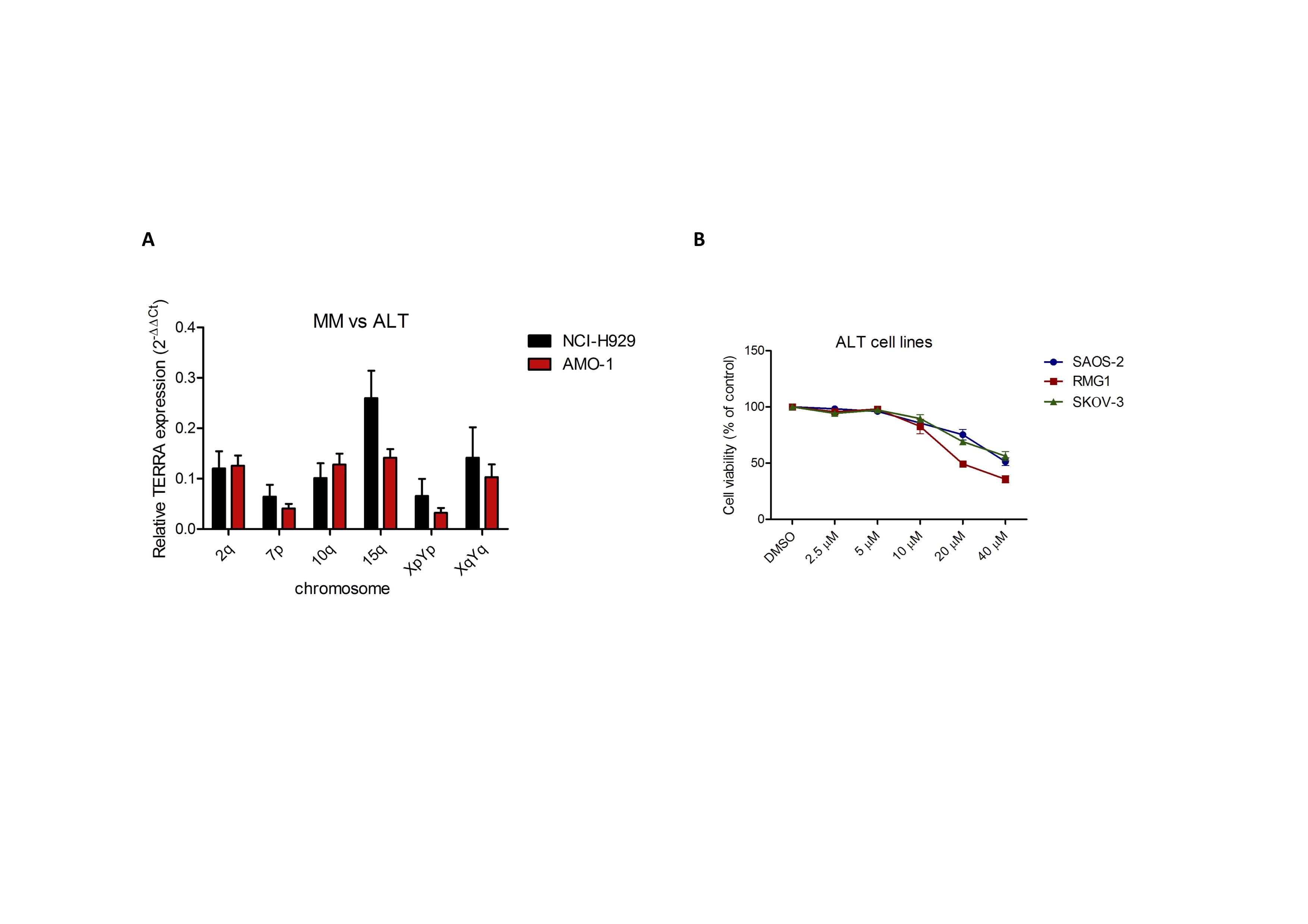

Supplement: Supplementary file 1 — Additional file 1: Figure. S1. A Basal TERRA levels in NCI-H929 and AMO-1 cell lines respect to three ALT cell lines (SAOS-2, SKOV-3 and RMG1) that usually display high levels of TERRA. B ALT cell lines were treated with hit 17 at 2.5, 5, 10 and 20 μM for 48 hours, followed by Cell Titer-Glo assay. [file 13046_2023_2633_MOESM1_ESM.jpg]

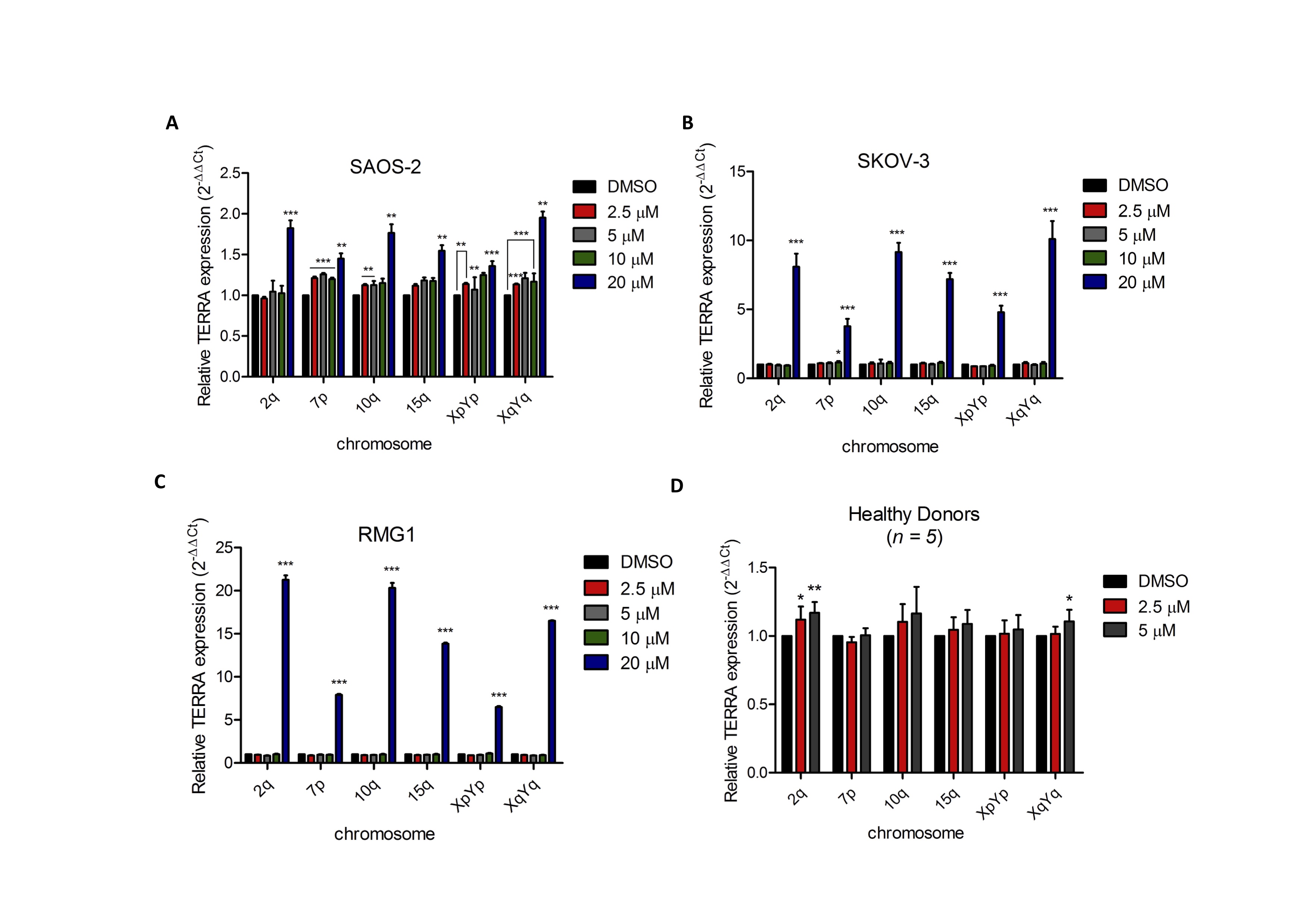

Supplement: Supplementary file 2 — Additional file 2: Figure. S2. A-C RT-qPCR of TERRAs after 48 hours of hit 17 treatments on SAOS-2, SKOV-3, and RMG1. D RT-qPCR of TERRAs after 48 hours of hit 17 treatments on PBMCs from five HD donors. * P<0.05; **P<0.01; *** P<0.001; **** P<0.0001. Results shown in B-D are the average of two independent biological replicates. Error bars are SD. [file 13046_2023_2633_MOESM2_ESM.jpg]

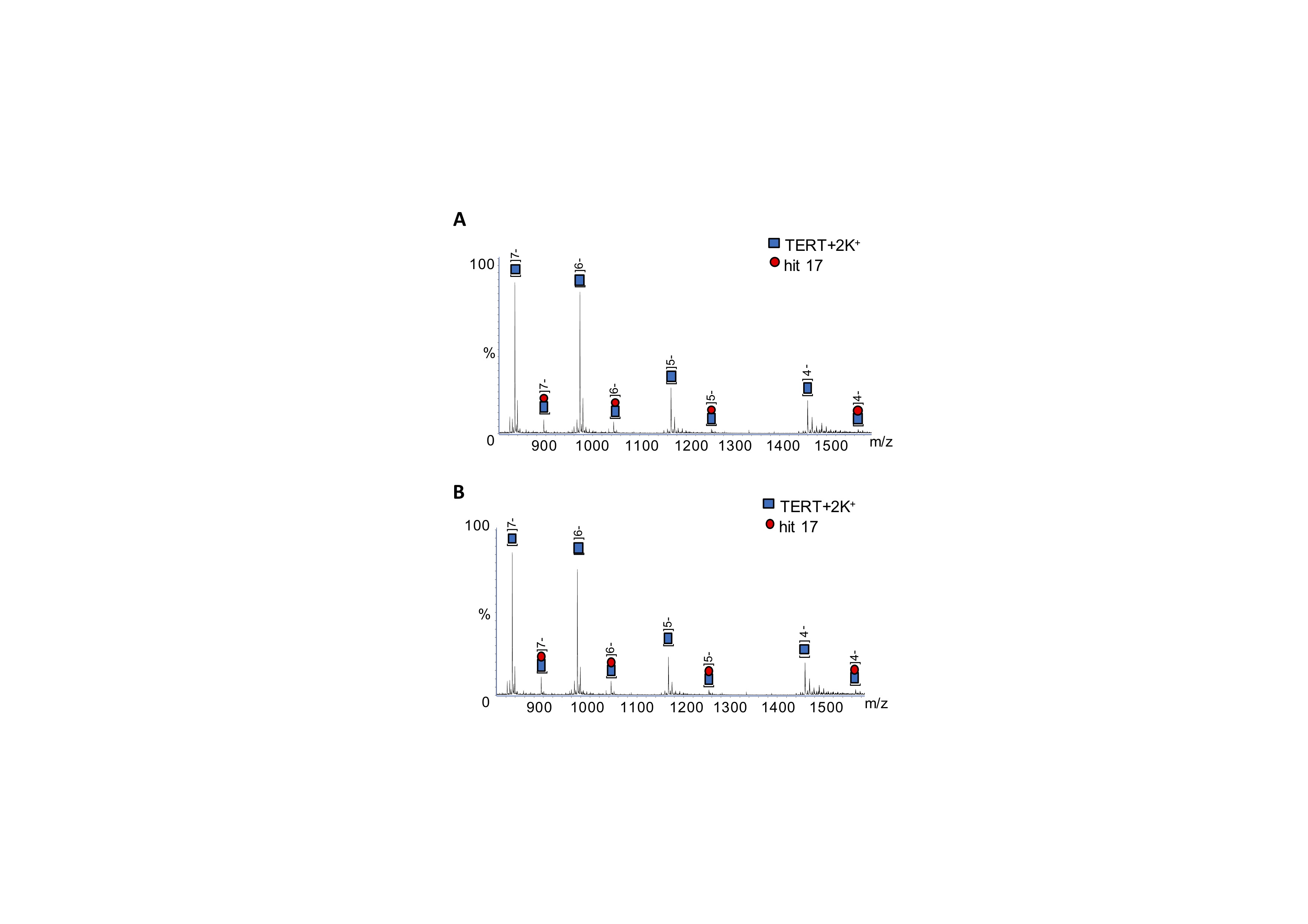

Supplement: Supplementary file 3 — Additional file 3: Figure. S3. A, B MS spectra of TERT (blue squares) incubated with hit 17 (red circles). Samples (5 μl) containing 5 μM of TERT oligonucleotide and 10 μM (panel A) or 20 μM (panel B) of hit 17 were incubated in MS buffer (HFIP 120 mM/TEA pH 7.4, KCl 0.8 mM, Isopropanol 20%) overnight before MS analysis. [file 13046_2023_2633_MOESM3_ESM.jpg]

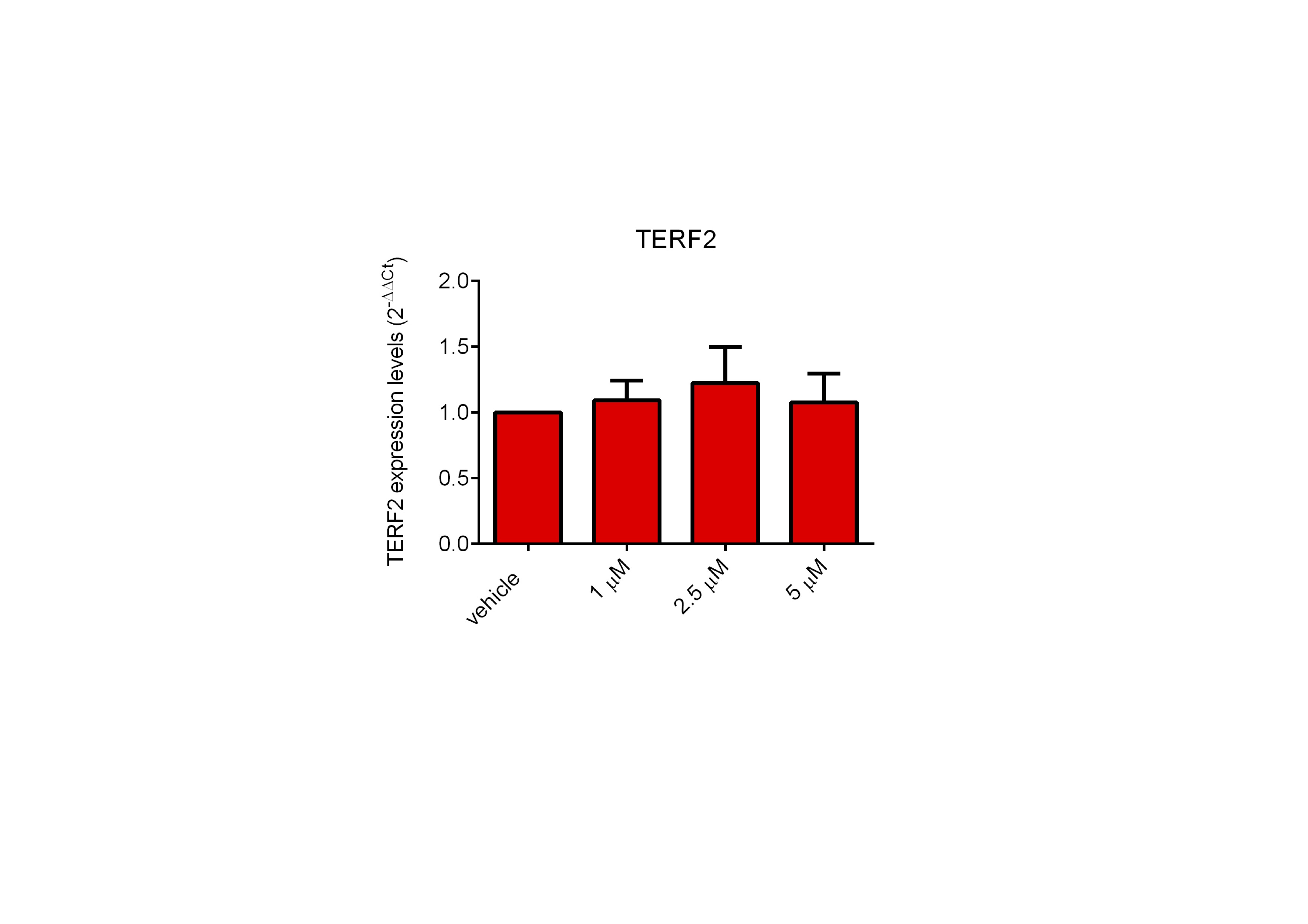

Supplement: Supplementary file 4 — Additional file 4: Figure. S4. RT-qPCR of TERF2 encoding TRF2 protein. Hit 17 treatments on the NCI-H929 cell line showed no modulation of the expression level of TRF2. [file 13046_2023_2633_MOESM4_ESM.jpg]

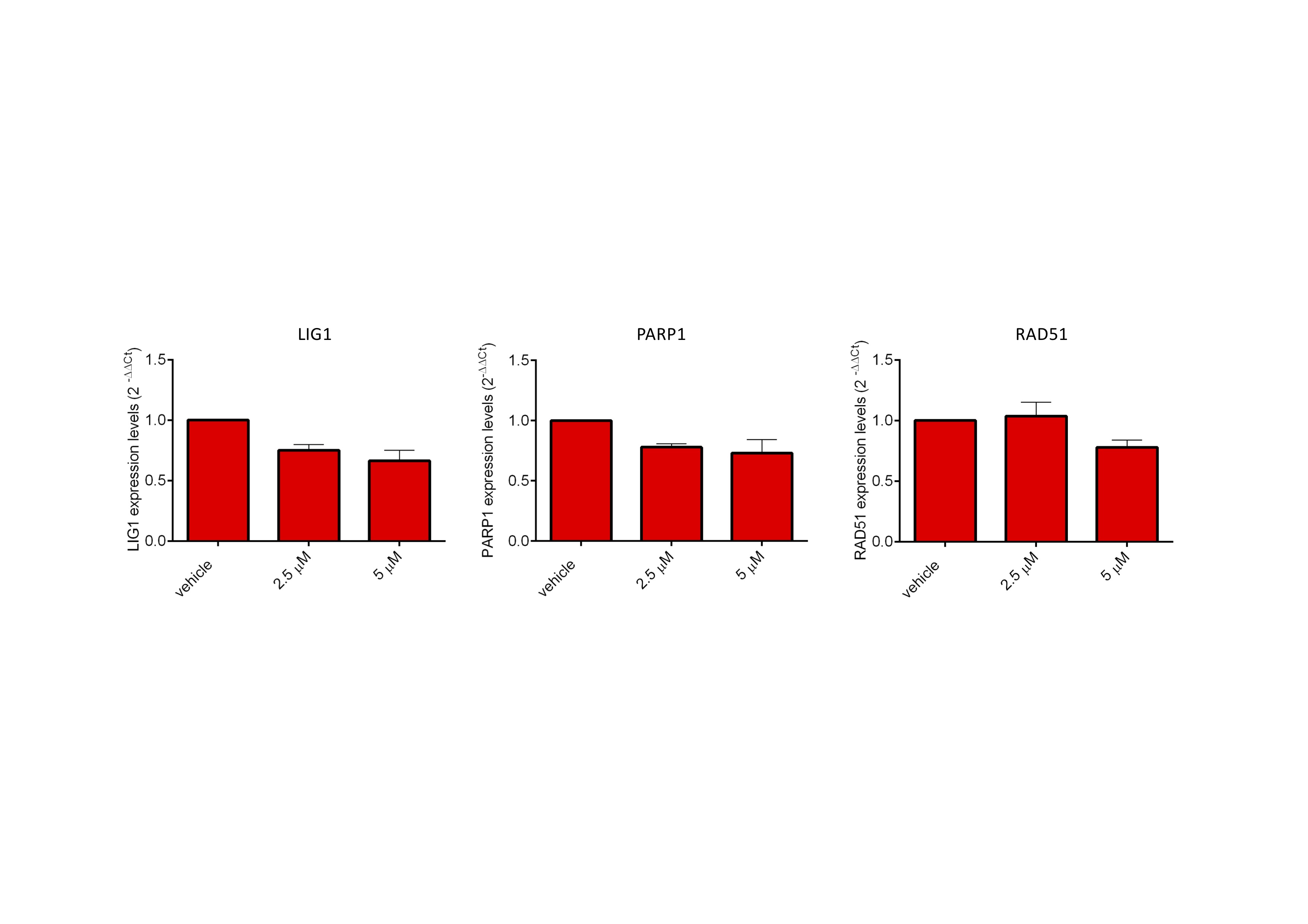

Supplement: Supplementary file 5 — Additional file 5: Figure. S5. Validation of DNA damage gene signature. RT-qPCR of selected genes (LIG1, PARP1, RAD51) involved in DNA damage response in the NCI-H929 cell line after hit 17 treatments. [file 13046_2023_2633_MOESM5_ESM.jpg]

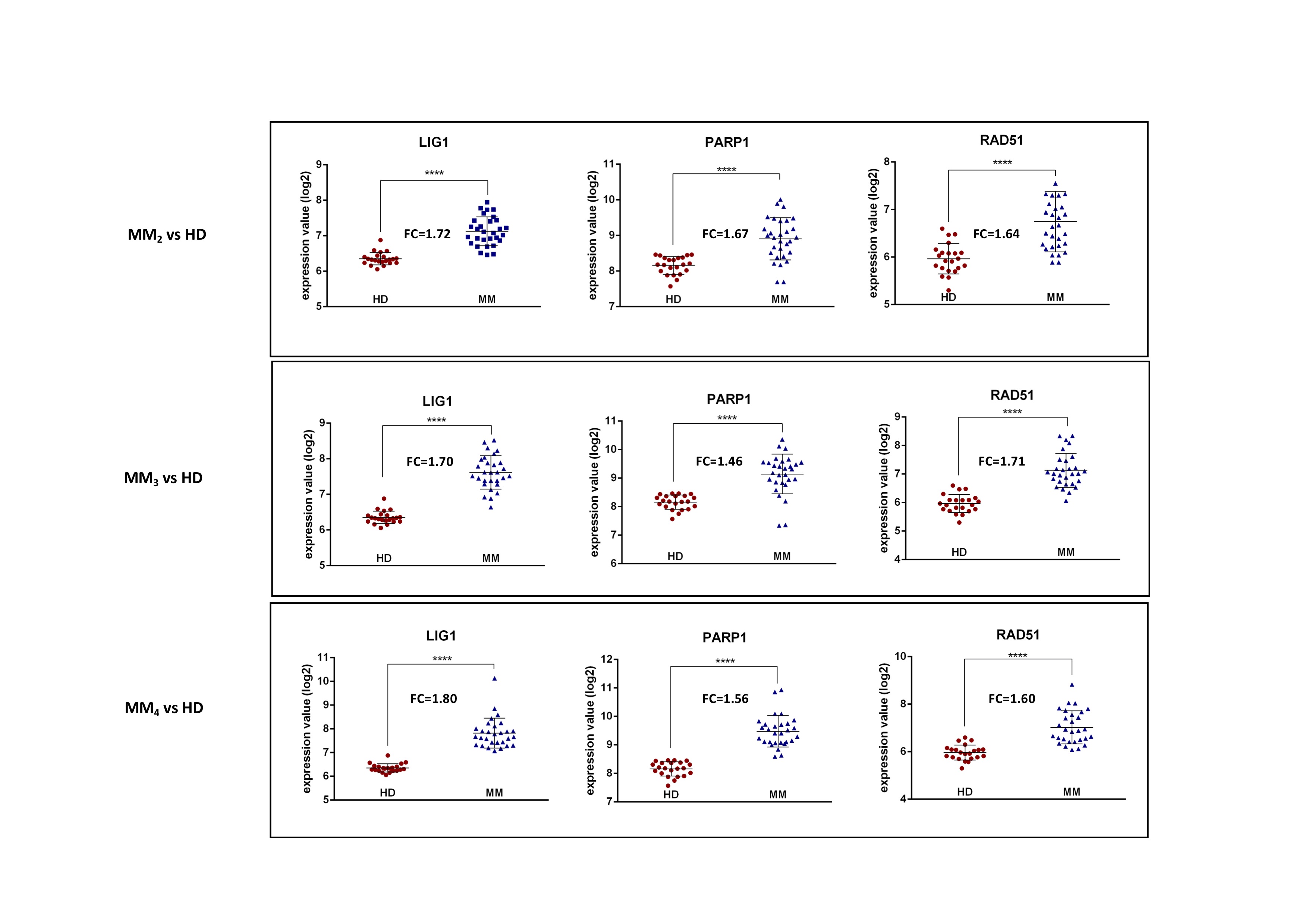

Supplement: Supplementary file 6 — Additional file 6: Figure. S6. DNA repair gene signature validation. Gene expression levels of LIG1, PARP1 and RAD51 in MM2, MM3, and MM4 validation cohorts from GSE19784 each compared to HD samples from GSE5900. FC= fold change. ****P<0.0001 [file 13046_2023_2633_MOESM6_ESM.jpg]
