# Supplementary material for: TERRA G-quadruplex stabilization as a new therapeutic strategy for multiple myeloma
Source: J Exp Clin Cancer Res. 2023 Mar 27;42:71. doi: 10.1186/s13046-023-02633-0 (PMC10041726; doi:10.1186/s13046-023-02633-0)
Supplement: Supplementary file 7 — Additional file 7: Table S1. List of significantly enriched gene sets performed on NCI-H929 with GSEA software. [file 13046_2023_2633_MOESM7_ESM.docx]

**Table S1. List of significantly enriched gene sets performed on NCI-H929 with GSEA software.**

| **Gene Set** | **NES** | **NOM** |
| --- | --- | --- |
| E2F_TARGETS | -4.39 | 0.00 |
| G2M_CHECKPOINT | -4.27 | 0.00 |
| MYC_TARGETS_V1 | -4.09 | 0.00 |
| OXIDATIVE_PHOSPHORYLATION | -3.87 | 0.00 |
| MITOTIC_SPINDLE | -3.54 | 0.00 |
| MYC_TARGETS_V2 | -3.53 | 0.00 |
| DNA_REPAIR | -3.34 | 0.00 |
| ADIPOGENESIS | -2.85 | 0.00 |
| FATTY_ACID_METABOLISM | -2.79 | 0.00 |
| REACTIVE_OXYGEN_SPECIES_PATHWAY | -2.59 | 0.00 |
| PEROXISOME | -2.58 | 0.00 |
| MTORC1_SIGNALING | -2.47 | 0.00 |
| GLYCOLYSIS | -2.40 | 0.00 |
| APICAL_JUNCTION | -2.36 | 0.00 |
| INTERFERON_ALPHA_RESPONSE | -2.34 | 0.00 |
| UV_RESPONSE_UP | -2.25 | 0.00 |
| SPERMATOGENESIS | -2.19 | 0.00 |
| PI3K_AKT_MTOR_SIGNALING | -2.10 | 0.00 |
| WNT_BETA_CATENIN_SIGNALING | -2.08 | 0.00 |
| ANDROGEN_RESPONSE | -1.99 | 0.00 |
| IL6_JAK_STAT3_SIGNALING | 0.74 | 0.88 |
| PANCREAS_BETA_CELLS | 0.84 | 0.66 |
| ANGIOGENESIS | 1.07 | 0.37 |
| KRAS_SIGNALING_UP | 1.14 | 0.23 |
| HEDGEHOG_SIGNALING | 1.24 | 0.20 |
| TGF_BETA_SIGNALING | 1.34 | 0.09 |
| EPITHELIAL_MESENCHYMAL_TRANSITION | 1.76 | 0.00 |
| APOPTOSIS | 1.86 | 0.00 |
| HYPOXIA | 2.10 | 0.00 |
| P53_PATHWAY | 2.34 | 0.00 |
| TNFA_SIGNALING_VIA_NFKB | 2.90 | 0.00 |
