# Supplementary material for: TERRA G-quadruplex stabilization as a new therapeutic strategy for multiple myeloma
Source: J Exp Clin Cancer Res. 2023 Mar 27;42:71. doi: 10.1186/s13046-023-02633-0 (PMC10041726; doi:10.1186/s13046-023-02633-0)
Supplement: Supplementary file 8 — Additional file 8: Table S2. Primer sequences. [file 13046_2023_2633_MOESM8_ESM.docx]

**Table S2. Primer sequences**

| **Primer name** | **Sequence (5’-3’)** |
| --- | --- |
| TERRA 2q  Fw  Rv | AAAGCGGGAAACGAAAAGC  GCCTTGCCTTGGGAGAATCT |
| TERRA 7p  Fw  Rv | CAATCTCGGCTCACCACAATC  GGAGGCTGAGGCAGGAGAA |
| TERRA 10q  Fw  Rv | AAAGCGGGAAACGAAAAGC  GCCTTGCCTTGGGAGAATCT |
| TERRA 15q  Fw  Rv | GCGTGGCTTTGGGACAACT  TGCAACCGGGAAAGATTTTATT |
| TERRA XpYp  Fw  Rv | GCGCGTCCGGAGTTTG  CCACAACCCCACCAGAAAGA |
| TERRA XqYq  Fw  Rv | GAAAGCAAAAGCCCCTCTGA  CCCCTTGCCTTGGGAGAA |
| Telomere  Fw  Rv | GGTTTTTGAGGGTGAGGGTGAGGGTGAGGGTGAGGGT  TCCCGACTATCCCTATCCCTATCCCTATCCCTATCCCTA |
| TS | AATCCGTCGAGCAGAGTT |
| ACX | GCGCGGCTTACCCTTACCCTTACCCTAACC |
