# Supplementary material for: TERRA G-quadruplex stabilization as a new therapeutic strategy for multiple myeloma
Source: J Exp Clin Cancer Res. 2023 Mar 27;42:71. doi: 10.1186/s13046-023-02633-0 (PMC10041726; doi:10.1186/s13046-023-02633-0)
Supplement: Supplementary file 9 — Additional file 9: Table S3. Samples from GSE19784 included and analyzed in this study. [file 13046_2023_2633_MOESM9_ESM.docx]

**Table S3. Samples from GSE19784 included and analyzed in this study**

| **MM_1_** | **MM_2_** | **MM_3_** | **MM_4_** |
| --- | --- | --- | --- |
| GSM493958 | GSM493988 | GSM494018 | GSM494048 |
| GSM493959 | GSM493989 | GSM494019 | GSM494049 |
| GSM493960 | GSM493990 | GSM494020 | GSM494050 |
| GSM493961 | GSM493991 | GSM494021 | GSM494051 |
| GSM493962 | GSM493992 | GSM494022 | GSM494052 |
| GSM493963 | GSM493993 | GSM494023 | GSM494053 |
| GSM493964 | GSM493994 | GSM494024 | GSM494054 |
| GSM493965 | GSM493995 | GSM494026 | GSM494055 |
| GSM493966 | GSM493996 | GSM494027 | GSM494056 |
| GSM493967 | GSM493997 | GSM494028 | GSM494057 |
| GSM493968 | GSM493998 | GSM494029 | GSM494058 |
| GSM493969 | GSM493999 | GSM494030 | GSM494059 |
| GSM493970 | GSM494000 | GSM494031 | GSM494060 |
| GSM493971 | GSM494001 | GSM494032 | GSM494061 |
| GSM493972 | GSM494002 | GSM494033 | GSM494062 |
| GSM493973 | GSM494003 | GSM494034 | GSM494063 |
| GSM493974 | GSM494004 | GSM494035 | GSM494065 |
| GSM493975 | GSM494005 | GSM494036 | GSM494066 |
| GSM493976 | GSM494006 | GSM494037 | GSM494067 |
| GSM493977 | GSM494007 | GSM494038 | GSM494068 |
| GSM493978 | GSM494008 | GSM494039 | GSM494069 |
| GSM493979 | GSM494009 | GSM494040 | GSM494070 |
| GSM493980 | GSM494010 | GSM494041 | GSM494071 |
| GSM493981 | GSM494011 | GSM494042 | GSM494072 |
| GSM493982 | GSM494012 | GSM494043 | GSM494073 |
| GSM493983 | GSM494013 | GSM494044 | GSM494074 |
| GSM493984 | GSM494014 | GSM494045 | GSM494075 |
| GSM493985 | GSM494015 | GSM494046 | GSM494076 |
| GSM493986 | GSM494016 | GSM494047 | GSM494077 |
| GSM493987 | GSM494017 | - | - |
